# Supplementary material for: Deletion of wfs1 Impairs Oligodendrocyte Precursor Cells Dorsal Distribution and Myelination Through the wfs1-hmgcs1 Axis in Zebrafish
Source: Biology (Basel). 2026 Aug 16;15(16):1402. doi: 10.3390/biology15161402 (PMC13510151; doi:10.3390/biology15161402)
Supplement: Supplementary file 1 [file biology-15-01402-s001.zip › File S1-Transcriptome sequencing data and differentially expressed genes (DEGs) in wfs1-- zebrafish at 5 dpf/1.GO/wfs1KOvsWT/ALL/wfs1KOvsWT.all_GObar.pdf]

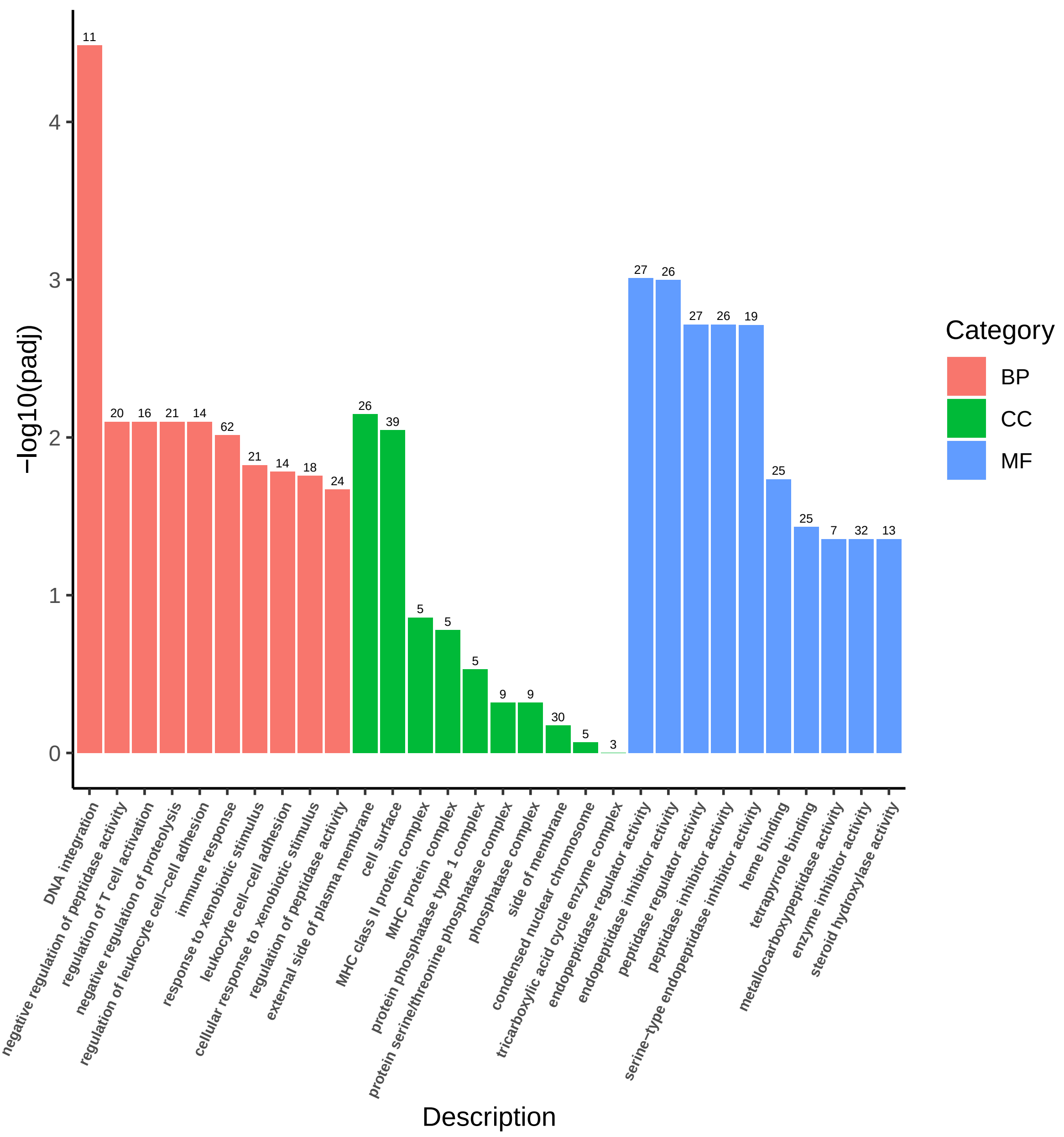

Category
BP
CC
MF
−log10(padj)
0
1
2
3
4
Description
11
20
16
21
14
62
21
14
18
24
26
39
5
5
5
9
9
30
5
3
27
26
27
26
19
25
25
7
32
13
DNA integration
negative regulation of peptidase activity
regulation of T cell activation
negative regulation of proteolysis
regulation of leukocyte cell−cell adhesion
immune response
response to xenobiotic stimulus
leukocyte cell−cell adhesion
cellular response to xenobiotic stimulus
regulation of peptidase activity
external side of plasma membrane
cell surface
MHC class II protein complex
MHC protein complex
protein phosphatase type 1 complex
protein serine/threonine phosphatase complex
phosphatase complex
side of membrane
condensed nuclear chromosome
tricarboxylic acid cycle enzyme complex
endopeptidase regulator activity
endopeptidase inhibitor activity
peptidase regulator activity
peptidase inhibitor activity
serine−type endopeptidase inhibitor activity
heme binding
tetrapyrrole binding
metallocarboxypeptidase activity
enzyme inhibitor activity
steroid hydroxylase activity
